# Glioblastoma-Derived Three-Dimensional Ex Vivo Models to Evaluate Effects and Efficacy of Tumor Treating Fields (TTFields)

**DOI:** 10.3390/cancers14215177

**Published:** 2022-10-22

**Authors:** Vera Nickl, Ellina Schulz, Ellaine Salvador, Laureen Trautmann, Leopold Diener, Almuth F. Kessler, Camelia M. Monoranu, Faramarz Dehghani, Ralf-Ingo Ernestus, Mario Löhr, Carsten Hagemann

**Affiliations:** 1 Section Experimental Neurosurgery, Department of Neurosurgery, University of Würzburg, 97080 Würzburg, Germany; 2 Department of Neuropathology, Institute of Pathology, University of Würzburg, 97080 Würzburg, Germany; 3 Department of Anatomy and Cell Biology, Martin-Luther-University Halle-Wittenberg, 06112 Halle (Saale), Germany

**Keywords:** glioblastoma, Tumor Treating Fields (TTFields), organotypic hippocampal slice cultures (OHSC), organoids, tumor slice cultures, 3D ex vivo models

## Abstract

**Simple Summary:**

In glioblastoma, tumor recurrence is inevitable and the prognosis of patients is poor, despite multidisciplinary treatment approaches involving surgical resection, radiotherapy and chemotherapy. Recently, Tumor Treating Fields (TTFields) have been added to the therapeutic set-up. These alternating electric fields are applied to glioblastoma at 200 kHz frequency via arrays placed on the shaved scalp of patients. Patients show varying response to this therapy. Molecular effects of TTFields have been investigated largely in cell cultures and animal models, but not in patient tissue samples. Acquisition of matched treatment-naïve and recurrent patient tissues is a challenge. Therefore, we suggest three reliable patient-derived three-dimensional ex vivo models (primary cells grown as microtumors on murine organotypic hippocampal slices, organoids and tumor slice cultures) which may facilitate prediction of patients’ treatment responses and provide important insights into clinically relevant cellular and molecular alterations under TTFields.

**Abstract:**

Glioblastoma (GBM) displays a wide range of inter- and intra-tumoral heterogeneity contributing to therapeutic resistance and relapse. Although Tumor Treating Fields (TTFields) are effective for the treatment of GBM, there is a lack of ex vivo models to evaluate effects on patients’ tumor biology or to screen patients for treatment efficacy. Thus, we adapted patient-derived three-dimensional tissue culture models to be compatible with TTFields application to tissue culture. Patient-derived primary cells (PDPC) were seeded onto murine organotypic hippocampal slice cultures (OHSC), and microtumor development with and without TTFields at 200 kHz was observed. In addition, organoids were generated from acute material cultured on OHSC and treated with TTFields. Lastly, the effect of TTFields on expression of the Ki67 proliferation marker was evaluated on cultured GBM slices. Microtumors exhibited increased sensitivity towards TTFields compared to monolayer cell cultures. TTFields affected tumor growth and viability, as the size of microtumors and the percentage of Ki67-positive cells decreased after treatment. Nevertheless, variability in the extent of the response was preserved between different patient samples. Therefore, these pre-clinical GBM models could provide snapshots of the tumor to simulate patient treatment response and to investigate molecular mechanisms of response and resistance.

## 1. Introduction

Glioblastoma (GBM) is one of the most aggressive primary brain tumors in adults with a median survival time of 16–18 months and a five-year survival rate of 6% for male and 9% for female patients [1]. During their limited lifespan, patients suffer from neurological deficits such as hemiparesis, aphasia, seizures and changes of personality, rendering the disease even more devastating [1,2]. Maximum standard therapy includes extensive surgery, if functionally possible, followed by radiotherapy combined with concomitant and adjuvant chemotherapy with temozolomide (TMZ) [3,4]. However, despite vigorous efforts in research during the last few years, relapse is unavoidable, and prognosis is even worse in patients with a multifocal involvement [3,5] or an unmethylated O^6^-methylguanine-DNA methyltransferase (MGMT) promoter [6,7]. MGMT is a repair enzyme counteracting TMZ chemotherapy [8].

Tumor Treating Fields (TTFields) therapy, which is applied via arrays placed onto the patient’s shaved scalp, and utilizes alternating electric fields with a frequency of 100–400 kHz and an intensity of 1–3 V/cm, is a new physical treatment modality for a diverse range of cancers [9,10]. Interference with the spindle apparatus and disturbance of cytokinesis, and thereby hindrance of mitosis and reduced cell proliferation, has been suggested as their primary mode of action [11,12,13,14]. In addition, several other effects caused by TTFields on the cellular level have been described [1,10,15]. The randomized multicenter EF-14 phase III trial proved the efficacy of TTFields at 200 kHz for the treatment of newly diagnosed GBM when applied with maintenance TMZ chemotherapy following maximal safe surgical resection and chemo-radiation [16]. The Kaplan-Meier curves from this trial imply that there are patients who respond very well to TTFields and other less responsive patients. However, it is still unknown which patients benefit the most from TTFields. Therefore, it might be crucial to identify patients who are likely to benefit from TTFields treatment prior to TTFields application. Since acquisition of matched treatment-naïve and recurrent patient tissues is a challenge, molecular effects of TTFields have been mainly investigated in cell cultures or animal models [15] and information on mechanisms leading to TTFields resistance is scarce. Thus, investigating how TTFields alter the tumor biology in humans is a so far insufficiently met necessity, and reliable organotypic ex vivo test systems, which would allow for a controlled and repeatable evaluation of the effects and efficacy of TTFields in the laboratory are critical.

In vitro models of various tumor entities, such as GBM, range from simple two-dimensional (2D) to the more complex three-dimensional (3D) systems. Two-dimensional models usually consist of one cell type supplemented with extracellular matrix proteins for support, but usually lack the representation of a tumor microenvironment (TME). Meanwhile, the more composite 3D models are customarily constructed to include one or more cell types cultured in a suitable scaffold made up of biomaterials [17]. Due to their complexity, they are able to resemble the in vivo TME much closer [18]. Some of the more recent 3D model systems available for GBM include glioma cell lines, GBM stem cell derived cultures, microtubes, human-induced pluripotent stem cells, organoids, organotypic slice cultures, as well as bioprinted chip systems [19]. Nonetheless, most of the 3D models that exist, even apart from those used for GBM, are mainly engineered for the purpose of drug screening [20,21,22]. None currently exist in combination with TTFields to screen for the aforementioned modalities including patient-suitability to predict maximum beneficial outcomes.

Consequently, we suggest three different patient-derived 3D ex vivo models for TTFields pre-clinical studies. Patient-derived material can be cultured and used as a representative surrogate to investigate TTFields function ex vivo. (1) GBM-patient-derived primary cells (PDPC) can be seeded onto murine organotypic hippocampal slice cultures (OHSC) to form microtumors [23]. (2) Organoids derived from intraoperatively resected material can be cultured up to several months, frozen and thawed while maintaining the tumor-inherent invasive, immunohistological, cellular and mutational profile [24]. For TTFields application, they are placed onto OHSC as a tissue carrier and grown as microtumors. (3) Organotypic tumor slice cultures generated from fresh intra-operative material that represents patient-specific tumor properties and the tumor microenvironment for a more realistic set-up, can be preserved up to 6 days [25].

The aim of the present study was to investigate whether the implementation of PDPC or patient-derived organoids, as well as organotypic tumor slice cultures, is technically feasible as respective 3D-model systems for testing the effect and efficacy of TTFields. This approach might hold future applications as a test system prior to TTFields treatment in order to determine patient response to therapy, optimize treatment such as testing possible combinations or optimal frequencies, and to investigate molecular mechanisms of treatment response and resistance.

## 2. Materials and Methods

### 2.1. Tissue Samples

All patients were newly diagnosed, did not receive any prior tumor treatment, and were operated at the Department of Neurosurgery, University Hospital Würzburg, Germany. They gave written informed consent in accordance with the declaration of Helsinki, and as approved by the Institutional Review Board of the University of Würzburg (#22/20-me). The histopathology of the tumor samples was confirmed by an experienced neuropathologist and classified according to the 2021 WHO criteria [26]. Only GBM, IDH-wildtype CNS WHO grade 4 samples were included.

### 2.2. Patient-Derived Primary Cells, Cell Lines, and Cell Culture

To generate PDPC, necrotic areas and blood vessels were removed from the intra-operatively obtained tumor tissue and the latter then separated using a homogenizer. The homogenized tumor material was cultured in 25 cm^3^ cell culture flasks (Corning, New York, NY, USA) in Dulbecco’s Modified Eagle’s Medium (DMEM) containing 1 g/L glucose, sodium pyruvate, 3.7 g/L NaHCO_3_ and L-glutamine and supplemented with 20% *v*/*v* heat-inactivated fetal calf serum (FCS), 2 × non-essential amino acids (NEAA, 100× stock, add 10 mL to 500 mL medium) (all from Gibco, Carlsbad, CA, USA) and 1.5% vitamin C (Sigma-Aldrich, St. Louis, MO, USA) at 37 °C, 5% CO_2_, and 95% humidity until an adherent cell layer was formed [27]. A GBM cell line U87MG (CLS, Eppelheim, Germany) was cultured in DMEM supplemented with 10% *v*/*v* FCS, 2 × NEAA, 3 mM L-glutamine, 100 U/mL penicillin, and 100 mg/mL streptomycin (Invitrogen, Carlsbad, CA, USA) as a monolayer under the same conditions.

### 2.3. Generation of Organoids

**Figure 1:**
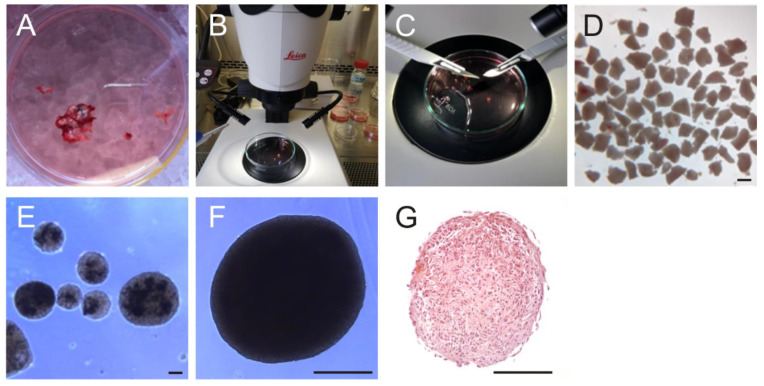
Preparation of GBM organoids. (**A**) Intra-operatively obtained tumor tissue was stored on ice in Hibernate A medium. (**B**–**D**) Under a stereo microscope, the tumor was minced into pieces of 0.5 mm, which were cultured. Scale bar = 0.5 mm. (**E**) The edges rounded up and organoids formed after two weeks. (**F**) Perfectly formed organoid. Organoids can be cultured for several weeks, propagated by mincing into 100 µm large pieces and re-grown or stored frozen in liquid nitrogen. (**G**) H/E staining of a cross-section through an organoid after 4 weeks of culture. Scale bars in (**E**–**G**) = 100 µm.

Organoids were prepared according to the method described by Jacob et al. [24]. Fresh intraoperatively obtained tumor tissue was temporarily stored on ice in Hibernate A medium (Gibco, Carlsbad, CA, USA) (Figure 1A). Next, the tissue was cleared of necrosis and blood vessels and carefully minced with a scalpel into approximately 0.5 mm pieces under the microscope (Figure 1B–D). These pieces were then treated with RBC Lysis Buffer (Invitrogen, Carlsbad, CA, USA) for 10 min and washed two times with Hibernate A medium containing 1% Glutamax, 0.4% penicillin/streptomycin and 0.1% Amphotericin (HGPSA) (all from Gibco, Carlsbad, CA, USA). Sections were transferred into GBO medium consisting of 47.24% DMEM/F12, 47.25% Neurobasal, 0.02% B27 without Vitamin A (50×), 0.01% Glutamax, 0.01% N2, 0.01% NEAA, 0.004% penicillin/streptomycin, 0.001% β-Mercaptoethanol (all from Gibco, Carlsbad, CA, USA) and 0.00023% human insulin (Sigma-Aldrich, St. Louis, MO, USA) to ultra-low attachment 6-well plates (Corning Costar, New York, NY, USA) and incubated at 37 °C, 5% CO_2_ and 95% humidity on an orbital shaker at 120 rpm. After 2 weeks of culture, organoids formed successfully and could be used for further experiments (Figure 1E–G).

### 2.4. Preparation of Organotypic Hippocampal Brain Slice Cultures (OHSC)

**Figure 2:**
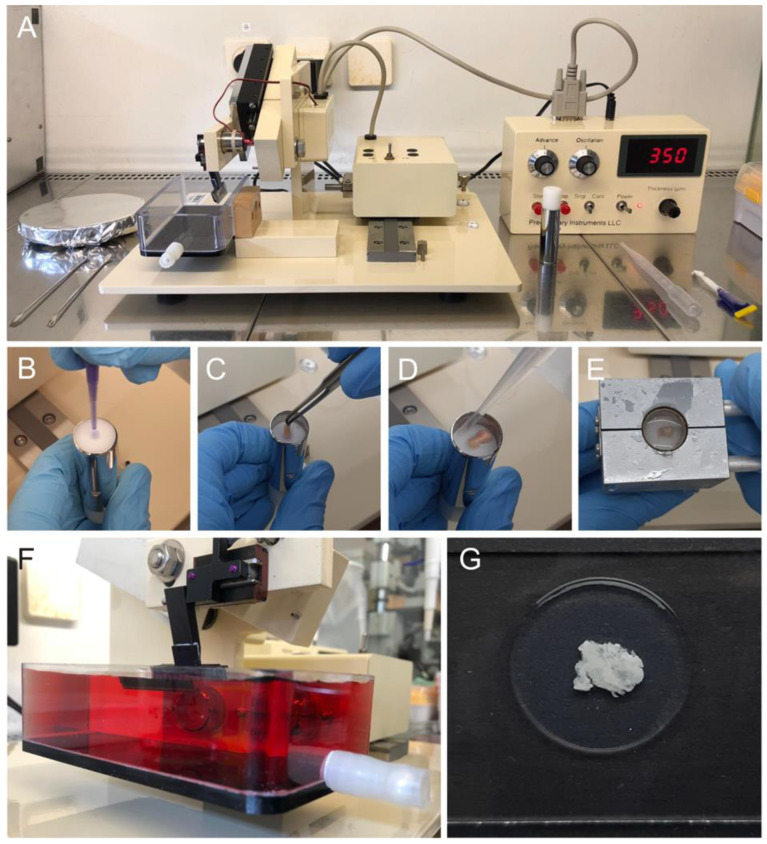
Preparation of organotypic hippocampal brain slice cultures (OHSC) and patient-derived tumor slices. (**A**) Vibratome and instruments required for the generation of tissue slices. (**B**) Histoacryl glue was placed onto the vibratome’s sample tube. (**C**) The brain and tumor, respectively, were glued on edge into the tube and (**D**) filled with molten agarose. (**E**) The agarose was hardened with a cooling block pre-cooled at −80 °C. (**F**) The vibratome was used to generate slices of 350 µm thickness. (**G**) The tumor slices were encased in agarose.

OHSC were prepared as described previously [23]. Briefly, mice 5–8 days postpartum (p5-p8) were decapitated following ethical guidelines. The brain was dissected under the microscope and glued with the dorsal surface facing downward into the sample tube of a vibratome (Precisionary Instruments, Greenville, SC, USA) (Figure 2A) with histoacryl glue (B. Braun, Tuttlingen, Germany) (Figure 2B,C). The tube was filled with molten agarose (Sigma-Aldrich, St. Louis, MO, USA) (Figure 2D). A cooling block pre-cooled at −80 °C and then stored on ice until use ensured rapid hardening of the agarose (Figure 2E). The sample tube was clamped into the vibratome, which was set to an advance of 3.5 and an oscillation of 6 and 350 µm thick slices were generated (Figure 2F). Slices were collected in a preparation tray filled with minimal essential medium (MEM) supplemented with 1% penicillin/streptomycin, 1% L-glutamine (all from Gibco, Carlsbad, CA, USA) and 1% glucose (Sigma-Aldrich, St. Louis, MO, USA). The hippocampus was isolated, transferred using a wide glass pipette and cultured on inserts with a semi-permeable membrane of 0.4 µm pore size (Greiner Bio-one, Frickenhausen, Germany) in a 24-well plate (Corning Costar, New York, NY, USA) containing brain slice medium (MEM supplemented with 25% normal horse serum, 25% Hank’s Balances salt solution (HBSS), 1% penicillin/streptomycin, 1% L-glutamine (all from Gibco, Carlsbad, CA, USA), vitamin C and 1% glucose (both from Sigma-Aldrich, St. Louis, MO, USA) at 35 °C, 5% CO_2_ and 95% humidity. It was vital that excess medium be aspirated from the OHSC. The slices obtain their nutrient supply from the medium via the pores of the insert and must not be covered by any liquid. In addition, the slice should be placed as centrally and planar as possible onto the insert. After a cultivation period of two weeks, experiments could be started.

### 2.5. Seeding of Fluorescence-Labeled GBM Cells and Organoids onto OHSC

The cells were fluorescently labeled for easy visualization of U87MG cells, PDPC and organoids growing on OHSCs. U87MG and PDPC were transfected with green fluorescent protein (GFP) utilizing the pmaxGFP plasmid (Lonza, Cologne, Germany) in combination with nucleofection using the Amaxa Cell Line Nucleofector Kit V (Lonza, Cologne, Germany), as detailed elsewhere [27,28]. Briefly, cells were detached with 0.25% trypsin/EDTA (Carl Roth, Karlsruhe, Germany) and suspended in cell culture medium. For each transfection, 1 × 10^6^ cells were centrifuged for 10 min at 300× *g* at room temperature. The supernatant was discarded, and the cells were re-suspended in 100 µL of Nucleofector solution V. In a 1.5 mL reaction tube, cells were mixed with 2 µg pmaxGFP plasmid, transferred to transfection cuvettes and electroporated with a transfection program U29. After transfection, cells were transferred to the wells of a 6-well plate containing 1 mL cell culture medium each. The cells were allowed to recover for 2–3 days before further use. Approximately 1 × 10^5^ cells were taken up in 10 µL cell culture medium and spread onto the surface of the OHSC. After 2–3 days, microtumor growth and invasion could be detected using an inverted fluorescence microscope LEICA DMI 3000 B (Leica, Wetzlar, Germany).

Organoids were mechanically minced with scalpels into pieces of 100 µm size and incubated in 10 µM Carboxyfluorescein succinimidyl ester (CFSE) in PBS for 15 min as provided in the CellTrace^TM^ CFSE Cell Proliferation Kit (Invitrogen, Carslbad, CA, USA) and following the manufacturer’s instructions. The solution was then replaced with fresh GBO medium and incubated at 37 °C for another 30 min. The next day, organoids fluoresced at an excitation wavelength of 488 nm and were placed onto the OHSC using a pipette.

### 2.6. Generation of Patient-Derived Organotypic Tumor Slice Cultures

Generation of organotypic tumor slice cultures was based on a publication by Merz et al. [25]. After surgical tumor resection, the tissue was directly transferred to Hibernate A medium and stored on ice. The tumor tissue was carefully freed from necrosis and blood vessels and cut into approximately 2 cm × 0.5 cm pieces using a scalpel. Preparation of slices was performed as described above for the OHSC (Figure 2). The slices were embedded in agarose (Figure 2G) and had to be carefully cut out with a scalpel before they could be transferred to the center of the semi-permeable membrane with a wide glass pipette. The vitality of slices, histopathology and tumor content were assessed by an experienced neuropathologist. Tumor slices could be cultured in brain slice medium at 35 °C, 5% CO_2_ and 95% humidity for up to 6 days. The medium was changed every second day.

### 2.7. TTFields Treatment

The inovitro™ laboratory research system (Novocure, Haifa, Israel) was used for TTFields administration. U87MG cells and PDPC cultured as monolayers were treated as described previously [29]. Briefly, glass coverslips with 20 mm diameter (Hartenstein, Würzburg, Germany) were placed into inovitro ceramic dishes (Novocure, Haifa, Israel). Cells were trypsinized and plated onto the coverslips by placing 350 µL cell culture medium containing 30,000 cells as a drop in their center. Cells attached during a 20 h incubation at 37 °C and 5% CO_2_. The medium was replaced by 2 mL fresh cell culture medium, the ceramic dishes were placed onto a base plate connected to a TTFields generator and TTFields at 200 kHz were applied with an intensity of 1.7 V/cm for 72 h. The medium was renewed every 48 h. Control cells were kept under the same conditions without TTFields application. To evaluate TTFields effects, cells were trypsinized and counted using the Scepter 2.1 cell counter (Merck, Darmstadt, Germany).

**Figure 3:**
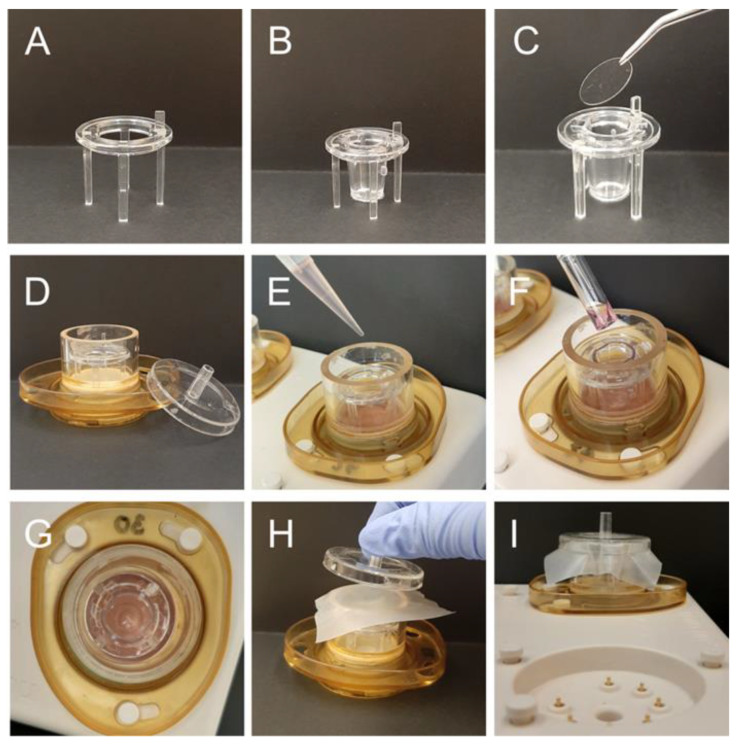
Sample preparation for TTFields treatment. (**A**) Holder for the inserts with semi-permeable membranes (0.4 µm pore size). (**B**) The insert was placed into the holder. (**C**) Glass coverslip (12 mm diameter) to protect the slices from condensation water. (**D**) The holder with the insert was placed into a ceramic dish with high walls. (**E**) The ceramic dish, but not the insert, was filled with 2.5 mL of brain slice medium. (**F**) An OHSC or tumor slice was transferred using a wide glass pipette and the excess medium was aspirated. (**G**) The slice should be centrally placed on the membrane of the insert. (**H**) Coverslip, parafilm and lid covered the ceramic dish which was (**I**) connected to the base plate for TTFields application.

To treat OHSC and tumor slices with TTFields, ceramic dishes with high walls (Novocure, Haifa, Israel) were utilized (Figure 3). The holders, containing the inserts with semi-permeable membranes, were placed into the dishes and 2.5 mL brain slice medium was pipetted into the dishes outside the inserts (Figure 3A–E). OHSC and tumor slices, respectively, were transferred as described above (Figure 3F,G). To avoid condensation water to drop onto the slice surfaces, a 12 mm coverslip was placed over each insert. The ceramic dish was covered with parafilm and closed with a lid to minimize evaporation of the medium (Figure 3C,H,I). TTFields were applied for 72 to 96 h at 200 kHz and 1.5 V/cm. Condensation water was carefully aspirated every day, and the medium was changed every second day. To evaluate microtumor growth on OHSC, images were taken using a LEICA DMI 3000 B microscope, LEICA DFC450 camera and LAS V4.5 software (all Leica, Wetzlar, Germany). Tumor size was determined on the fluorescence images using the Measure Tool from the open-source program Fiji (Image J 1.53c) [30,31]. Tumor slices were fixed in 4% formalin (Carl Roth, Karlsruhe, Germany) for 24 h at 4 °C and then transferred to phosphate buffered saline (PBS) (Sigma-Aldrich, St. Louis, MO, USA) for immunohistochemical and immunofluorescence staining.

### 2.8. Immunohistochemical and Immunofluorescence Staining

The fixed brain slices were dehydrated, embedded in paraffin, and sliced into 3 µm thick sections. Next, standardized hematoxylin and eosin (HE) (Carl Roth, Karlsruhe, Germany) staining was performed for histology. For immunohistochemical staining, Ki67 (ab16667, Abcam, Cambridge, UK) and GFAP (sc33673, santacruz, Dallas, TX, USA) antibodies were used at a dilution of 1:1000 and 1:100 in antibody dilution buffer (DCS Innovative Diagnostik Systeme, Hamburg, Germany), respectively, and incubated overnight at 4 °C. Protein expression was visualized using the secondary antibodies AlexaFluor488 and AlexaFluor555 (both from Invitrogen, Carlsbad, CA, USA) in a 1:1000 dilution, and incubated for 1 h at room temperature. Finally, the slices were mounted using Fluoroshield mounting medium containing DAPI (Abcam, Cambridge, UK). Five representative fields of view per slide were photographed with the LEICA DMI 3000 B microscope with standardized settings at 40× magnification and analyzed for staining intensity via the batch processing function of the open-source program Fiji (ImageJ 1.53c) [30,31]. The macro settings are described elsewhere [32].

### 2.9. Statistical Analysis

Statistical analysis was performed using GraphPadPrism 9 software (GraphPad Software, San Diego, CA, USA). Statistical significance was defined by unpaired 2-tailed *t*-tests, and by ANOVA. *p* < 0.05 was considered to be significant. In the box plots the boxes represent the median with the 25% and 75% quartile and the whiskers the minimum and maximum of the data set. Descriptive statistics were obtained to calculate the tumor size and its growth or decrease. The mean value was expressed as percentage with standard error of the mean (±SEM). The difference was also shown with ± SEM as well as the 95% confidence interval (CI). All experiments were performed at least in triplicates.

## 3. Results

### 3.1. Patient Cohort

To establish the described ex vivo GBM models, tumor samples of eight GBM patients were utilized (Table 1). The tumors were classified according to the most recent WHO classification of tumors of the central nervous system [26]. Only GBM, IDH-wildtype CNS WHO grade 4 were used. A low amount of tumor material prevented execution of all models with the same samples in this proof of principle study.

### 3.2. Patient-Derived GBM Primary Cells Display Variable Responses to TTFields at 200 kHz

**Figure 4:**
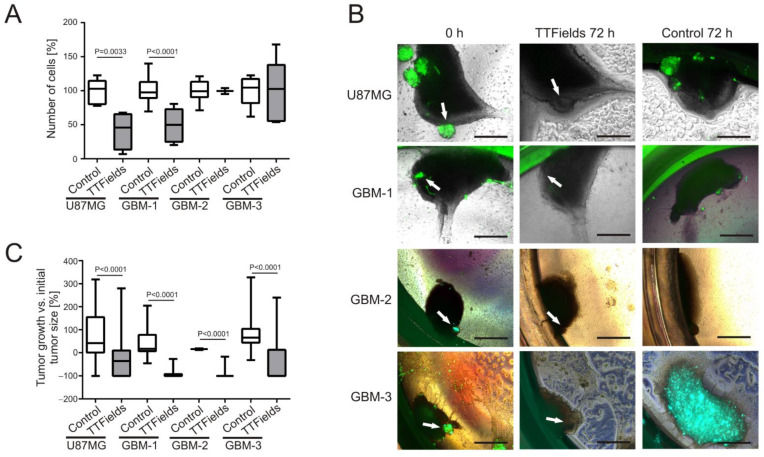
Variable responses of patient-derived GBM primary cells to TTFields treatment. (**A**) Cells were grown as monolayer, treated with TTFields at 200 kHz for 72 h and counted (*n* = 3–18). (**B**) Cells were seeded onto organotypic hippocampal slice cultures (OHSC) and grew to microtumors within 3 days (0 h) as visualized by fluorescence microscopy (green). Control cells remained untreated for 72 h (Control 72 h), whereas TTFields-treated cells were subjected to TTFields at 200 kHz for 72 h (TTFields 72 h). Arrows point to microtumors before TTFields application, and the area of former microtumor location in the same OHSC after 72 h TTFields application. Scale bar = 250 µm. Shown are representative images of U87MG (*n* = 24), GBM-1 (*n* = 7), GBM-2 (*n* = 7), and GBM-3 (*n* = 13). (**C**) Quantification of the microtumor growth on OHSC. A minimum of 3 and up to 83 microtumors were measured. For clinical details of the patient derived-primary cells refer to Table 1.

Previously published data revealed that application of TTFields at 200 kHz significantly decreased the number of GBM cells [11,12,29,33]. In our experiments, we treated U87MG cells and three PDPC monolayer cell cultures with TTFields at 200 kHz for 72 h. These cells responded to TTFields to a variable extent (Figure 4A). Whereas the cell numbers of U87MG cells were reduced to 41.75% ± 13.76% compared to the untreated control (*p* = 0.0033, CI = −90.76 to −25.74) and those of GBM-1 were diminished to 49.7% ± 10.87% (*p* < 0.0001, CI = −69.19 to −31.41), GBM-2 and GBM-3 were non-responsive to TTFields treatment (Figure 4A).

We seeded these cells onto OHSC to investigate whether a 3-dimensional growth in the form of microtumors could reproduce these results. Three days after seeding, microtumors had formed (Figure 4B) and were subjected to TTFields at 200 kHz for 72 h. The response of U87MG and GBM-1 microtumors, respectively, was confirmed (Figure 4B,C). During the 72 h observation period, U87MG microtumors in the no-treatment control grew on average by 72.84% ± 18.01%. In contrast, TTFields led to shrinkage of microtumors by −28.59% ± 7.72%, which is a difference between the averages of −101.4 ± 16.72 percentage points (*p* < 0.0001, CI = −134.6 to −68.29) (Figure 4C). Similarly, GBM-1 microtumors grew by 36.5% ± 19.07% if not treated with TTFields, whilst the treated microtumors downsized by −90.37% ± 4.82%, a difference of −126.9 ± 16.19 percentage points (*p* < 0.0001, CI = −160.0 to −93.75) (Figure 4C). Surprisingly, GBM-2 and GBM-3 microtumors were responsive to TTFields, when grown in form of microtumors. GBM-2 microtumors grew by 15.5% ± 3.5% when they remained untreated, but shrank −90.72% ± 6.37% when subjected to TTFields, a difference of −106.2 ± 19.58 percentage points (*p* < 0.0001, CI = −147.4 to −65.09). The response of GBM-3 was very similar. Control microtumors grew by 100.6% ± 27.57%, while TTFields-treated tumors were reduced in size by −37.64% ± 14.64%, which is a difference of −138.2 ± 28.78 percentage points (*p* < 0.0001, CI = −196.1 to −80.4) (Figure 4C). We did not observe any detrimental effects of TTFields at 200 kHz on the OHSC carrier tissue.

### 3.3. TTFields Induce Shrinkage of Patient-Derived GBM-Organoids Growing on OHSC

**Figure 5:**
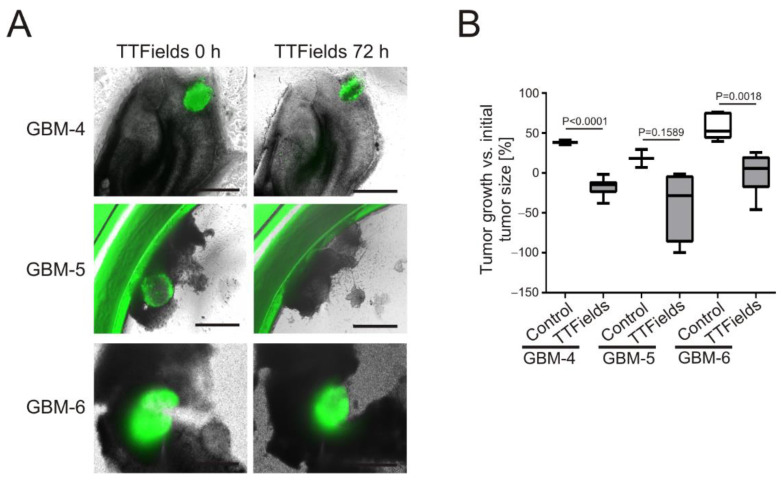
Patient-derived GBM-organoid size is reduced by TTFields treatment. (**A**) Comparison of different patient-derived GBM organoids grown on organotypic hippocampal slice cultures (OHSC) before (TTFields 0 h) and after treatment with TTFields at 200 kHz for 72 h (TTFields 72 h). Organoids were visualized by fluorescence microscopy (green). Shown are representative images of *n* = 6. Scale bar = 250 µm. (**B**) Quantification of the organoid-growth on OHSC. A minimum of three and up to six, organoids were measured. For clinical details of the patient-derived organoids refer to Table 1.

The above data indicate that GBM cells growing as 3D microtumors exhibit an increased sensitivity towards TTFields. Therefore, to be even closer to the tumor tissue-like three-dimensional structure, organoids generated from fresh, intra-operatively gained GBM tissue of three different patients were grown on OHSC as the carrier tissue. The organoids of all patients grew into the OHSC within 2–3 days but displayed a heterogeneous appearance and growth pattern after TTFields treatment at 200 kHz for 72 h (Figure 5A). GBM-4 organoids were decreased in size by −17.10% ± 3.07%, whereas the untreated control organoids grew by 38.0% ± 3.0%. This was a difference in the average organoid size of −55.10 ± 7.21 percentage points (*p* < 0.0001, CI = −71.17 to −39.03). Although the organoids of GBM-5 clearly shrank by −39.68% ± 3.0% under TTFields treatment, the difference of −57.76 ± 33.41 percentage points to the control organoids, which grew by 18.08% ± 11.30%, was not statistically significant (*p* = 0.1589, CI = −150.5 to 35.01). In contrast, TTFields-treated GBM-6 organoids did not shrink, but were stalled in their growth (−0.003% ± 10.55%), whereas control organoids grew by 58.12% ± 7.13%. This was a statistically significant difference of −58.12 ± 13.32 percentage points (*p* = 0.0018, CI = −88.26 to −27.98) (Figure 5B).

### 3.4. Cell Proliferation Decreases and Apoptosis Increases in Human Organotypic GBM Tumor Slice Cultures when Treated with TTFields

**Figure 6:**
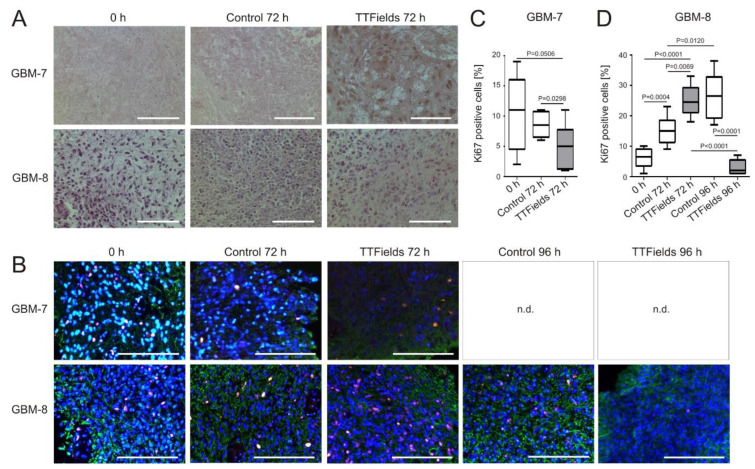
Decreased proliferation of TTFields-treated human organotypic GBM tumor slice cultures. Human GBM were sliced, cultured, treated with TTFields at 200 kHz for the indicated time periods and (**A**) hematoxylin and eosin-stained. (**B**) Immunofluorescence staining of adjacent slices of the same tumors for Ki67 (red), GFAP (green) and with DAPI (blue). Shown are representative images of *n* = 3. Scale bar = 100 µm. n.d. = not determined. (**C**) Quantification of Ki67 positive cells in slice cultures of GBM-7 and (**D**) GBM-8 after treatment with TTFields at 200 kHz. For clinical details of the patient-derived tumor slices refer to Table 1.

Human organotypic GBM tumor slice cultures were treated with TTFields at 200 kHz for 72 h. HE staining revealed a general decrease in cell number and a concomitant increase of apoptotic cells in the treated slices compared to untreated controls (Figure 6A). While staining for the proliferation marker Ki67 remained basically constant over time in GBM-7 (mean expression 10.38% ± 2.14% at 0 h vs. 8.63% ± 0.71% 72 h later), and only slightly dropped to 5.0% ± 1.32% (*p* = 0.0298, CI −6.84 to −0.41) when TTFields were applied (Figure 6B,C), GBM-8 displayed a completely different picture. There was an increase of Ki67 positivity by 9.17 ± 1.994 percentage points from 6% ± 1.99% (0 h) to 15.17% ± 2.01% (72 h) observable not only in untreated control slices (*p* = 0.0004, CI 4.89 to 13.44), but even more pronounced by 19.0 ± 2.05 percentage points to 25.0% ± 2.1% after 72 h TTFields treatment (*p* < 0.0001, CI 14.61 to 23.39) (Figure 6B,D). Ki67 staining only identifies cell cycle entry and does not reveal if the cell cycle will be completed [34,35]. Since TTFields have been shown to induce G1 cell cycle arrest [36], we wondered whether this increase in Ki67 would be sustained, and incubated the GBM-8 slices for another 24 h with TTFields. At 96 h total treatment, the percentage of Ki67 positive cells within the slice dropped to 3.0% ± 1.14% (*p* < 0.0001, CI −27.74 to −16.26), while Ki67 rose to 26.5% ± 3.12% in control cells (*p* = 0.0120, CI 3.09 to 19.57) (Figure 6B,D).

## 4. Discussion

In drug screening, effects of potential candidates are initially tested using in vitro models before embarking on a more costly, albeit more specific and detailed, in vivo study. Although in vitro systems are efficient and entail simple procurement, they often fail to demonstrate physiological interactions that occur in vivo. Nonetheless, even though the employment of in vivo methods more closely resembles clinical settings, humans and animals still differ in responses [37]. Therefore, the use of 3D tumor models such as spheroids and organoids has provided an opportunity to perform in vitro experiments using materials obtained from patient tissue, bridging the gaps between in vitro, pre-clinical and clinical set-ups. The use of spheroids and organoids eliminate the limitation of two-dimensional 2D models and are more consistent with in vivo studies, since they can contain several cell types such as stromal cells [17,18]. Moreover, they can be developed to resemble the tumor microenvironment more closely, allowing functional investigations of drug responses as well as metastasis, such as combining the 3D model with biomaterials [17], or engineering them to mimic physiological environments by using tumor-on-a-chip technology [38]. Three-dimensional models have been successfully used for drug screening studies for breast, lung and colon cancer [20,21,22]. Studies in GBM using 3D systems are also not far behind, as more research favoring them emerge [19,39,40]. However, none of these models has ever been combined with TTFields treatment ex vivo.

TTFields added to the standard maintenance therapy have improved the median progression-free and overall survival of newly diagnosed GBM patients by 2.7 and 4.9 months, respectively. Above that, they more than doubled the 5-year survival from 5% to 13% [16]. TTFields are mostly well tolerated, with systemic toxicity for the TTFields plus standard treatment group being comparable to the standard therapy group, with mild to moderate skin toxicity, e.g., skin rash and eczema underneath the arrays, occurring in 52% of patients in the TTFields group [16,41]. In order to reach highest efficacy, TTFields should be applied for ≥18 h each day on average [42]. It has been discussed that carrying the device, which weighs 1.3 kg, on a daily basis might restrict patients from daily activities and might hamper social life [43]. In addition, to place the arrays, patients need to shave their scalps, which could lead to stigmatization [43]. However, preliminary data from the recent TIGER trial do not support such apprehensions [44]. However, TTFields hardware is costly and maintenance is expensive [10]. Moreover, not all patients respond to TTFields to the same extent; some gain only a slight advantage, while others survive for 5 years or longer [16]. A standard sub-group analysis based on, e.g., MGMT promoter methylation status, extent of resection, Karnofsky performance index (KPI), or age, failed to identify which patients respond better to TTFields, as a survival benefit was demonstrated in all sub-groups [16]. Thus, patients who benefit from TTFields should be chosen wisely to allocate TTFields treatment efficiently. Information on mechanisms leading to TTFields resistance is very limited, and most molecular effects of TTFields have been investigated in cell cultures or animal models only [15]. Hence, the analysis of patient tumor samples before and after TTFields treatment would be desirable. However, we know from our own clinical experience that a bias could exist amongst such patients. Their TTFields device usage might vary [42], as well as the total treatment duration [16]. In addition, the time between end of the TTFields treatment and occurrence of relapse differs from patient to patient. Finally, patients with a reduced KPI might not be re-operated upon, while the time until relapse and following re-surgery might be prolonged in other patients. These factors could limit the availability of tissue for research. On the other hand, tumor samples of especially these patient groups are most interesting in terms of investigating treatment resistance related to molecular and cellular differences of inter-patient tumor heterogeneity and post-therapeutical changes.

To address these limitations, we tested several patient-derived ex vivo tumor tissue culture methods to identify patients who might benefit from TTFields therapy prior treatment, and to investigate short- and long-term effects of TTFields, including molecular prerequisites leading to TTFields responsiveness or resistance. At first, the standard GBM cell line U87MG and different PDPC monolayer cultures were treated with TTFields. As expected, there was a high variability of TTFields response, with some PDPC not reacting at all, possibly reflecting the patients’ tumor sensitivity for TTFields treatment. However, when grown as microtumors on OHSC, the cells became more sensitive. Thus, three-dimensional growth might better represent the in vivo situation and lead to more reliable results, as also has been shown for other cancer entities, such as breast cancer [20]. Nevertheless, while this primary cell-only approach might be easily done from a technical point of view, it has restricted validity as a screening system for clinical application. Due to the rupture of cell-cell-contacts during lysis, duration of cultivation, lack of tumor microenvironment, hypoxic gradients, and medium components such as serum, PDPC cultures not only changes their antigen surface expression patterns, but also undergoes molecular and transcriptional changes and thus no longer represent the parental tumor characteristics [19,24,39]. New ex vivo models such as organoids or tumor slice cultures might overcome these hurdles [17,18,19,39,40].

Organoids represent the histological characteristics, cellular diversity, gene expression, and mutational profiles of their corresponding parental tumors, as was successfully demonstrated for breast, lung and colon cancer, as well as GBM [18,20,21,22,40,45]. In addition, they can be generated quickly and reliably within two weeks from intraoperatively gained tissue [24]. Thus, they would be available for TTFields testing already two weeks after surgery, and results of TTFields screenings could be expected at a time when patients completed radiotherapy and could start with TTFields application. Our data show that organoids grown on OHSC respond to TTFields treatment by interpatient heterogeneous appearance and growth patterns, with TTFields causing shrinkage of the microtumors to varying extents. Thus, patient-derived GBM tumor organoids represent an ideal and flexible model to not only test personalized therapies by correlating mutational profiles with responses to TTFields, but also to investigate changes in organoid cell structures and molecular protein expression patterns caused by TTFields. Since organoids can be propagated over long time periods without changing their properties [24], they also should be suitable to investigate long-term TTFields effects in a patient related setting.

In addition to the above-mentioned paternal specifics, patient derived organotypic tumor slice cultures retain the tumor microenvironment, including neural cells, and therefore are even closer to the in vivo tumor situation of GBM patients [25]. Freshly sliced, the tissue is ready to use, but on the downside, slicing is a delicate method highly dependent on tissue quality, and is susceptible to deficiencies. Handling these cultures requires a high level of experience, and they are viable only for a couple of days. On the other hand, adjacent slices from the same tumor region can be generated and cultured for better comparison of different experimental conditions. Especially, molecular alterations can be visualized in slice cultures. As a proof of concept, we stained slice cultures for Ki67, a well-established proliferation marker in pathological evaluation of tumor tissue [35]. It was intriguing that the percentage of Ki67 positive cells in one of the investigated GBM samples increased over time despite TTFields treatment, which was expected to interfere with cell proliferation [11,12,13]. However, Ki67 staining identifies cells which have entered the cell cycle and is high in G1, S, G2 and M phase, but does not give an indication about the cells’ later fate [34,35]. TTFields can cause cell cycle arrest, which might lead to accumulation of Ki67-positive cells for a certain time. Indeed, when staining the slices after 96 h instead of 72 h treatment, there was a massive drop in Ki67 positivity, probably due to Ki67 degradation and cell death. While this observation is based on only one single tumor and should not be overinterpreted, it proves the feasibility of investigating TTFields-induced molecular changes in cultured tumor tissue slices. Therefore, both organoids and tumor slice cultures have different advantages, which complement each other.

Taken together, the screening methods we describe demonstrate high feasibility for use in patients who are being considered for TTFields treatment. As has been suggested by Gilazieva et al., it is advisable to combine different methods and model systems to obtain reliable results when studying tumor response to therapies [18]. The same 3D systems may likely apply to other future treatment modalities in which the same concept would prove beneficial. Nonetheless, our focus is to provide clinicians an aid in discerning the likelihood of patients to be good candidates for TTFields therapy, for proper allocation.

Like every other study, ours has some limitations. First, the ex vivo data were not matched with the clinical data of the respective patients. Second, we performed only a small number of experiments for each system. As the main goal of this study was to prove the technical feasibility of combining patient derived organotypic ex vivo culture systems with TTFields, we now plan to continue our studies by matching experimental data with the clinical course of the patients in a prospective bench to bedside and back approach.

## 5. Conclusions

In this small study we were able to identify differences in the inter-patient treatment response not only when using PDPC cultures, but especially when utilizing patient-derived organoids grown on OHSC and tumor slice cultures. This establishes our methodology as a powerful tool to screen for patients that might benefit from TTFields treatment, as well as to elucidate cellular alterations within the cultured tissue. Last but not least, these models will shed light onto molecular mechanisms of treatment response and resistance, especially if they are used in combination.

## 6. Patents

U.S. Provisional Patent Application No. 63/409,525 is based on the work reported in this manuscript.

## Figures and Tables

**Table 1:** Clinical parameters of glioblastoma samples and the models they were utilized for.

ID	Sex	Age [Years]	Histology	KPI	Ki67 [%]	MGMT Promoter Methylation [%]	IDH1 Mutation	IDH2 Mutation	ATRX Expression	Experiment
1	male	64	GBM	100	20	yes (76)	no	no	yes	PDPC
2	female	79	GBM	100	20	yes (66)	no	no	yes	PDPC
3	female	73	GBM	100	50	yes (28)	no	no	yes	PDPC
4	female	69	GBM	30	25	yes (71)	no	no	yes	organoid
5	female	65	GBM	90	20	no (8.0)	no	no	yes	organoid
6	male	65	GBM	90	40	no (4.0)	no	no	yes	organoid
7	male	64	GBM	40	30	yes (82)	no	no	yes	tumor slice
8	male	54	GBM	90	40	no (3)	no	no	yes	tumor slice

ID = identification number; KPI = Karnofsky performance index; MGMT = O^6^-methylguanine-DNA methyltransferase; IDH = isocitrate dehydrogenase; ATRX = α thalassemia/mental retardation syndrome X-linked; GBM = glioblastoma; PDPC = patient derived primary cells.

## Data Availability

All data are contained within the manuscript. Raw data are available on reasonable request from the corresponding author.

## References

[1] Wick W. (2021). Gliome, S2k-Leitlinie. *Leitlinien für Diagnostik und Therapie in der Neurologie*. Deutsche Gesellschaft für Neurologie, Ed. www.dgn.org/leitlinien.

[2] Fritz L., Dirven L., Reijneveld J.C., Koekkoek J.A.F., Stiggelbout A.M., Pasman H.R.W., Taphoorn M.J.B. (2016). Advance care planning in glioblastoma patients. Cancers.

[3] Stupp R., Mason W.P., van den Bent M.J., Weller M., Fisher B., Taphoorn M.J.B., Belanger K., Brandes A.A., Marosi C., Bogdahn U. (2005). Radiotherapy plus concomitant and adjuvant temozolomide for glioblastoma. N. Engl. J. Med..

[4] Stupp R., Hegi M.E., Mason W.P., van den Bent M.J., Taphoorn M.J.B., Janzer R.C., Ludwin S.K., Allgeier A., Fisher B., Belanger K. (2009). Effects of radiotherapy with concomitant and adjuvant temozolomide versus radiotherapy alone on survival in glioblastoma in a randomized phase III study: 5-year analysis of the EORTC-NCIC trial. Lancet Oncol..

[5] Patil C.G., Eboli P., Hu J. (2012). Management of multifocal and multicentric gliomas. Neurosurg. Clin. N. Am..

[6] Esteller M., Garcia-Foncillas J., Andion E., Goodman S.N., Hidalgo O.F., Vanaclocha V., Baylin S.B., Herman J.G. (2000). Inactivation of the DNA-repair gene MGMT and the clinical response of gliomas to alkylating agents. N. Engl. J. Med..

[7] Rivera A.L., Pelloski C.E., Gilbert M.R., Colman H., De La Cruz C., Sulman E.P., Bekele B.N., Aldape K.D. (2010). MGMT promoter methylation is predictive of response to radiotherapy and prognostic in the absence of adjuvant alkylating chemotherapy for glioblastoma. Neuro Oncol..

[8] Feldheim J., Kessler A.F., Monoranu C.M., Ernestus R.-I., Löhr M., Hagemann C. (2019). Changes of O6-methylguanine DNA methyltransferase (MGMT) promotor methylation in glioblastoma relapse—A meta-analysis type literature review. Cancers.

[9] Carieri F.A., Smack C., Siddiqui I., Kleinberg L.R., Tran P.T. (2020). Tumor treating fields: At the crossroad between physics and biology for cancer treatment. Front. Oncol..

[10] Rominiyi O., Vanderlinden A., Clenton S.J., Bridgewater C., Al-Tamimi Y., Collis S.J. (2020). Tumour treating fields therapy for glioblastoma: Current advances and future direction. Br. J. Cancer.

[11] Kirson E.D., Gurvich Z., Schneiderman R., Dekel E., Itzhaki A., Wasserman Y., Schatzberger R., Palti Y. (2004). Disruption of cancer cell replication by alternating electric fields. Cancer Res..

[12] Kirson E.D., Dbalý V., Tovarys F., Vymazal J., Soustiel J.F., Itzhaki A., Mordechovich D., Steinberg-Shapira S., Gurvich Z., Schneiderman R. (2007). Alternating electric fields arrest cell proliferation in animal tumor models and human brain tumors. Proc. Natl. Acad. Sci. USA.

[13] Riley M.M., San P., Lok E., Swanson K.D., Wong E.T. (2019). The clinical application of tumor treating fields therapy in glioblastoma. J. Vis. Exp..

[14] Moser J.C., Salvador E., Deniz K., Swanson K., Tuszynski J., Carlson K.W., Karanam N.K., Patel C.B., Story M., Lou E. (2022). The mechanisms of action of Tumor Treating Fields. Cancer Res..

[15] Kissling C., Di Santo S. (2020). Tumor Treating Fields—Behind and beyond inhibiting the cancer cell. CNS Neurol Disord Drug Targets.

[16] Stupp R., Tallibert S., Kanner A., Read W., Steinberg D., Lhermitte B., Toms S., Idbaih A., Ahluwalia M.S., Fink K. (2017). Effect of Tumor-Treating Fields plus maintenance temozolomide vs. maintenance temozolomide alone on survival in patients with glioblastoma: A randomized clinical trial. JAMA.

[17] Nii T., Makino K., Tabata Y. (2020). Three-dimensional culture system of cancer cells combined with biomaterials for drug screening. Cancers.

[18] Gilazieva Z., Ponomarev A., Rutland C., Rizvnov A., Solovyeva V. (2020). Promising applications of tumor spheroids and organoids for personalized medicine. Cancers.

[19] Paolillo M., Comincini S., Schinelli S. (2021). In vitro glioblastoma models: A journey into the third dimension. Cancers.

[20] Imamura Y., Mukohara T., Shimono Y., Funakoshi Y., Chayahara N., Toyoda M., Kiyota N., Takao S., Kono S., Nakatsura T. (2015). Comparison of 2D- and 3D-culture models as drug-testing platforms in breast cancer. Oncol. Rep..

[21] Nii T., Makina K., Tabata Y. (2019). A cancer invasion model combined with cancer-associated fibroblasts aggregates incorporating gelatin hydrogel microspheres containing a p53 inhibitor. Tissue Eng. Part C Methods.

[22] Goudar V.S., Koduri M.P., Ta Y.-N.N., Chen Y., Chu L.-A., Lu L.-S., Tseng F.-G. (2021). Impact of a desmoplastic tumor microenvironment for colon cancer drug sensitivity: A study with 3D chimeric tumor spheroids. ACS Appl. Mater Interfaces.

[23] Schulz E., Hohmann T., Hohmann U., Ernestus R.-I., Löhr M., Dehghani F., Hagemann C. (2021). Preparation and culture of organotypic hippocampal slices for the analysis of brain metastasis and primary brain tumor growth. Methods Mol. Biol..

[24] Jacob F., Salinas R.D., Zhang D.Y., Nguyen P.T.T., Schnoll J.G., Wong S.Z.H., Thokala R., Sheikh S., Saxena D., Prokop S. (2020). A patient-derived glioblastoma organoid model and biobank recapitulates inter- and intra-tumoral heterogeneity. Cell.

[25] Merz F., Gaunitz F., Dehghani F., Renner C., Meixensberger J., Gutenberg A., Giese A., Schopow K., Hellwig C., Schäfer M. (2013). Organotypic slice cultures of human glioblastoma reveal different susceptibilities to treatments. Neuro Oncol..

[26] Louis D.N., Perry A., Wesseling P., Brat D.J., Cree I.A., Figarella-Branger D., Hawkins C., Ng H.K., Pfister S.M., Reifenberger G. (2021). The 2021 WHO classification of tumors of the central nervous system: A summary. Neuro Oncol..

[27] Hagemann C., Amend D., Kessler A.F., Linsenmann T., Ernestus R.-I., Löhr M. (2017). High-efficiency transfection of glioblastoma cells and a simple spheroid migration assay. Methods Mol Biol.

[28] Hagemann C., Meyer C., Stojic J., Eicker S., Gerngras S., Kühnel S., Roosen K., Vince G.H. (2006). High efficiency transfection of glioma cell lines and primary cells for overexpression and RNAi experiments. J. Neurosci. Methods.

[29] Kessler A.F., Frömbling G.E., Gross F., Hahn M., Dzokou W., Ernestus R.-I., Löhr M., Hagemann C. (2018). Effects of tumor treating fields (TTFields) on glioblastoma cells are augmented by mitotic checkpoint inhibition. Cell Death Discov..

[30] Schindelin J., Arganda-Carreras I., Frise E., Kaynig V., Longair M., Pietzsch T., Preibisch S., Rueden C., Saalfeld S., Schmid B. (2012). Fiji: An open-source platform for biological-image analysis. Nat. Methods.

[31] Schneider C.A., Rasband W.S., Eliceiri K.W. (2012). NIH Image to ImageJ: 25 years of image analysis. Nat. Methods.

[32] Feldheim J., Kessler A.F., Schmitt D., Wilczek L., Linsenmann T., Dahlmann M., Monoranu C.M., Ernestus R.-I., Hagemann C., Löhr M. (2018). Expression of activating transcription factor 5 (ATF5) is increased in astrocytomas of different WHO grades and correlates with survival of glioblastoma patients. Onco. Targets Ther..

[33] Giladi M., Schneiderman R.S., Voloshin T., Porat Y., Munster M., Blat R., Sherbo S., Bomzon Z., Urman N., Itzhaki A. (2015). Mitotic spindle disruption by alternating electric fields leads to inproper chromosome segregation and mitotic catastrophe in cancer cells. Sci. Rep..

[34] Thomasova D., Anders H.-J. (2015). Cell cycle control in the kidney. Nephrol. Dial. Transplant..

[35] Li L.T., Jiang G., Chen Q., Zheng J.N. (2015). Ki67 is a promising molecular target in the diagnosis of cancer (Review). Mol. Med. Rep..

[36] Voloshin T., Munster M., Blatt R., Shteingauz A., Roberts P.C., Schmelz E.M., Giladi M., Schneiderman R.S., Zeevi E., Porat Y. (2016). Alternating electric fields (TTFields) in combination with paclitaxel are therapeutically effective against ovarian cancer cells in vitro and in vivo. Int. J. Cancer.

[37] Saeidnia S., Manayi A., Abdollahi M. (2015). From in vitro experiments to in vivo and clinical studies; pros and cons. Curr. Drug Discov. Technol..

[38] Wan L., Neumann C.A., LeDuc P.R. (2020). Tumor-on-a-chip for integrating a 3D tumor microenvironment: Chemical and mechanical factors. Lab Chip.

[39] Joseph J.V., Blaavand M.S., Daubon T., Kruyt F.A.E., Thomsen M.K. (2021). Three-dimensional culture models to study glioblastoma—Current trends and future perspectives. Curr. Opin. Pharmacol..

[40] Orchestron-Findlay L., Bax S., Utama R., Engel M., Govender D., O’Neill G. (2021). Advanced spheroid, tumouroid and 3D bioprinted in-vitro models of adult and paediatric glioblastoma. Int. J. Mol. Sci..

[41] Lacouture M.E., Davis M.E., Elzinga G., Butowski N., Tran D., Villano J.L., Di Meglio L., Davies A.M., Wong E.T. (2014). Characterization and management of dermatologic adverse events with the novoTTF-100A system, a novel anti-mitotic electric field device for the treatment of recurrent glioblastoma. Semin. Oncol..

[42] Toms S.A., Kim C.Y., Nicholas G., Ram Z. (2019). Increased compliance with tumor treating fields therapy is prognostic for improved survival in the treatment of glioblastoma: A subgroup analysis of the E-14 phase III trial. J. Neurooncol..

[43] Kwan K., Schneider J.R., Boockvar J.A. (2018). Quality of life in patients with glioblastoma treated with tumor- treating fields. JAMA.

[44] Bähr O., Tabatabai G., Fietkau R., Goldbrunner R., Glas M. (2022). First results of the TIGER study—Treatment decision and quality of life of glioblastoma patients during TTFields therapy in routine clinical care. Proceedings of the Jahrestagung der Deutschen Gesellschaft für Neurochirurgie (DGNC), Joint Meeting mit der Griechischen Gesellschaft für Neurochirurgie.

[45] Xu X., Li L., Luo L., Shu L., Si X., Chen Z., Xia W., Huang J., Liu Y., Shao A. (2021). Opportunities and challenges of glioma organoids. Cell Commun. Signal.

